# Influence of Boundary Layer Structure and Low-Level Jet on PM_2.5_ Pollution in Beijing: A Case Study

**DOI:** 10.3390/ijerph16040616

**Published:** 2019-02-20

**Authors:** Yucong Miao, Shuhua Liu, Li Sheng, Shunxiang Huang, Jian Li

**Affiliations:** 1 State Key Laboratory of Severe Weather, Chinese Academy of Meteorological Sciences, Beijing 100081, China; miaoyucong@yeah.net (Y.M.); lijian08@pku.edu.cn (J.L.); 2 Department of Atmospheric and Oceanic Sciences, School of Physics, Peking University, Beijing 100871, China; lshuhua@pku.edu.cn; 3 Numerical Weather Prediction Center of China Meteorological Administration, Beijing 100081, China; 4 Institute of Chemical Defense, Beijing 102205, China

**Keywords:** low-level jet, PM_2.5_ pollution, mountain-plain breeze, planetary boundary layer

## Abstract

Beijing experiences frequent PM_2.5_ pollution, which is influenced by the planetary boundary layer (PBL) structure/process. Partly due to a lack of appropriate observations, the impacts of PBL on PM_2.5_ pollution are not yet fully understood. Combining wind-profiler data, radiosonde measurements, near-surface meteorological observations, aerosol measurements, and three-dimensional simulations, this study investigated the influence of PBL structure and the low-level jet (LLJ) on the pollution in Beijing from 19 to 20 September 2015. The evolution of the LLJ was generally well simulated by the model, although the wind speed within the PBL was overestimated. Being influenced by the large-scale southerly prevailing winds, the aerosols emitted from the southern polluted regions could be easily transported to Beijing, contributing to ~68% of the PM_2.5_ measured in Beijing on 20 September. The relative contribution of external transport of PM_2.5_ to Beijing was high in the afternoon (≥80%), which was related to the strong southerly PBL winds and the presence of thermally-induced upslope winds. On 20 September, the LLJ in Beijing demonstrated a prominent diurnal variation, which was predominant in the morning and after sunset. The occurrence of the LLJ could enhance the dilution capacity in Beijing to some extent, which favors the dilution of pollutants at a local scale. This study has important implications for better understanding the complexity of PBL structure/process associated with PM_2.5_ pollution in Beijing.

## 1. Introduction

**Figure 1:**
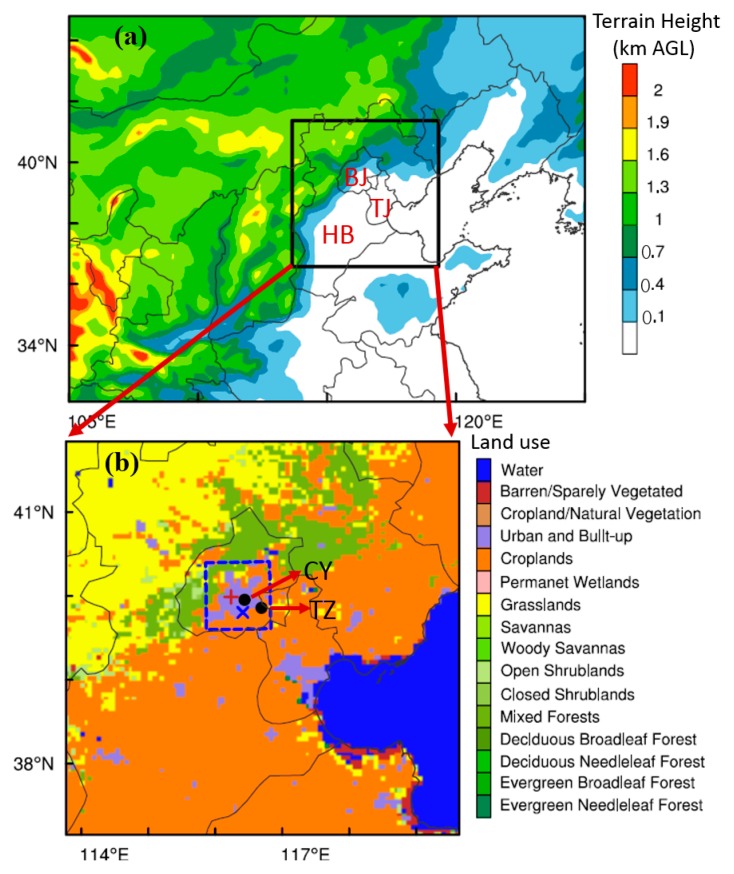
(**a**) Map of terrain height in simulation domains, and (**b**) land use categories in the inner domain. The black square in (**a**) denotes the locations of inner domain, and the blue dashed square in (**b**) indicates the region of interest (ROI) using the zero-out emission configuration. The red plus sign, blue cross, and black dots in (**b**) indicate the locations of wind-profiler, radiosonde site, and surface meteorological stations, respectively. The locations of Beijing, Tianjin, and Hebei are indicated by the red texts “BJ”, “TJ”, and “HB” in (**a**). The PM_2.5_ concentrations are also measured at those surface meteorological stations in Beijing, including Chaoyang (CY) and Tongzhou (TZ). AGL: above ground level.

The capital of China, Beijing, is located at the northern tip of the North China Plain (Figure 1a) and covers ~16, 410 km^2^, with a population greater than 21 million. Due to the rapid development of economy and the increase in energy usage over recent decades, heavy aerosol pollution events have frequently occurred in Beijing [1,2,3,4,5], characterzed by high PM_2.5_ concentration. From 2008 to 2014, the annual average PM_2.5_ concentration in Beijing varied between ~91 and 105 μg m^−3^, with the highest hourly average PM_2.5_ (≥990 μg m^−3^) recorded on 23 January 2012 [6]. Therefore, great effort has been put into investigating the PM_2.5_ pollution [7,8,9,10,11]. It has been found that the pollution in Beijing is not only caused by the high local emissions and chemical reaction/formation, but is also modulated by the meteorological factors [12,13,14,15]. Under certain unfavorable synoptic conditions, the secondary aerosol formation could contribute to ~70% of PM_2.5_ in Beijing [2].

The PM_2.5_ pollution events in Beijing are often found to be associated with southerly winds, high relative humidity (RH), and a shallow planetary boundary layer (PBL) [5,10,15,16]. With mountains to the north and west of Beijing (Figure 1a), the thermally induced mountain-plain breeze circulations develop frequently and modulate the PBL structure and PM_2.5_ pollution [12,14,17]. The mountain-plain breeze circulation is generally produced by the thermal contrast induced by the warming/cooling of mountains [18,19]. During the daytime, the upslope breeze can transport pollutants from urban regions toward the mountainside [9,20]. From the seasonality perspective, the seasonal variations in PBL thermal conditions and mountain–plain breeze circulation are critical to the seasonal changes in pollution in Beijing [14,15], which are partly responsible for the frequent occurrence of haze there in fall and winter. Compared with other seasons, in fall the local PBL process/structure plays a more important role in modulating the air quality in Beijing [14].

Another atmospheric phenomenon associated with the PBL structure, which may impact the pollution levels, is the low-level jet (LLJ). The LLJ is a narrow channel of relatively fast-travelling winds in the lower troposphere [19,21]. A strong wind shear below the jet can induce turbulence between the surface and the jet maximum level (i.e., jet nose), and influence the transport of heat, energy, moisture, and pollutants [22,23,24]. The LLJ occurs more frequently after sunset, which is related to inertial oscillation of the ageostrophic winds due to the sudden decay of turbulence after sunset within the PBL [25,26]. Compared with the relatively extensive studies of mountain–plain breeze in Beijing, the characteristics of the LLJ and their impact on pollution there are rarely investigated, partly due to the lack of appropriate observations. Therefore, this study aims to investigate the PBL structure associated with LLJ and the impacts on PM_2.5_ pollution in Beijing using wind-profiler observations and three-dimensional simulations. 

The rest of this paper is organized as follows. In Section 2, the observational data and design of numerical experiments are described. In Section 3, the simulation results are first validated against the observations, and then the influences of the LLJ and PBL structure on PM_2.5_ pollution in Beijing are examined based on the model outputs. Finally, the main findings are summarized in Section 4.

## 2. Data and Methods

### 2.1. Observational Data and Episode Description

The wind-profiler deployed to Beijing (116.28 °E, 39.98 °N, illustrated by the red plus sign in Figure 1b) is the CFL-16 profiler [24], which provides 25 levels of wind speed and direction below ~3 km above ground level (AGL) with a vertical resolution of 120 m. The detailed specifications of the wind-profiler are given in Table 1. Prior to the analysis, the wind-profiler data were strictly controlled for data consistency, continuity and deviation [24,27]. In this study, the LLJ was identified according to the maximum wind speed at the nose and decrease in speed above the jet nose [28,29]. An LLJ profile typically has a maximum wind speed greater than or equal to 10 ms^−1^ below 3 km AGL, and the decrease in wind speed from the jet to 3 km AGL is at least 5 ms^−1^. Similar LLJ definitions have been widely used in previous studies [24,27,30]. In addition to the wind-profiler measurements, radiosonde soundings in Beijing (116.47 °E, 39.80 °N, marked by the blue cross in Figure 1b) were also collected. The sounding balloon was launched twice a day at ~08:00 and 20:00 h Beijing time (BJT = UTC + 8h).

On the ground level, hourly 2-m temperature, RH, and PM_2.5_ concentrations were collected from two sites (marked by the black dots in Figure 1b) in Beijing, including the Tongzhou site (116.76 °E, 39.85 °N) and Chaoyang site (116.50 °E, 39.95 °N). The measurements of PM_2.5_ mass concentration at these two sites were properly conducted following the China Environmental Protection Standards, and the uncertainty of PM_2.5_ concentration was less than 5 μg m^−3^. All these measurements mentioned were taken regularly in September 2015.

The selected pollution episode in Beijing occurred on 20 September 2015, and there was no precipitation during the studied period. As shown by the MODIS true image and aerosol optical depth (AOD) in Figure S1, the value of AOD in Beijing was less than 0.2 on 19 September, while many aerosols were found in southern Hebei (Figure S1a,c). Then, thick aerosol plumes were observed in Beijing on 20 September, which were extended from southern Hebei (Figure S1b,d). Such a day-to-day change of aerosol concentration in Beijing is hypothesized to be induced by the transport of pollutants from the southern Hebei, which will be examined using three-dimensional simulations in Section 3.

### 2.2. Numerical Simulation

In this study, the Weather Research and Forecasting model coupled with Chemistry (WRF-Chem version 4.0, which is currently maintained by the Mesoscale and Microscale Meteorological Division of National Center for Atmospheric Research, Boulder, CO, USA) was used, which can simulate the transport, mixing, and chemical formation of gases and aerosols simultaneously with meteorological fields [31]. Two one-way nested domains (Figure 1a) were set using the MODIS land-use data of 2012, with horizontal grid spacing of 15 and 5 km. The inner domain assumed Beijing was the domain center, and covered the majority of the Beijing–Hebei–Tianjin region (Figure 1b). The model was initialized at 20:00 h BJT on 17 September, and run for 76 h until to 00:00 h BJT on 21 September. The first 28 h were considered as a spin-up period. In the vertical dimension, 48 vertical layers were set, extending from the surface to the 100-hPa level, with 26 layers below 3 km AGL to better resolve the PBL processes and structures. The initial and boundary conditions of meteorological variables were set using the 6-h 1° × 1° National Center for Environment Prediction (NCEP) global final (FNL) reanalysis. The physics parameterization schemes used included: the Lin microphysics scheme [32], the RRTMG longwave/shortwave radiation scheme [33], the YSU PBL scheme [34], and the Noah land surface scheme [35]. The PBL height of the YSU scheme was determined by using the bulk Richardson number approach [34]. In addition, to simulate the variations in aerosol concentration, the RADM2-MADE/SORGAM [36,37,38] mechanism was employed using the Multi-resolution Emission Inventory of China of 2012 (MEIC, illustrated in Figure S2), which is one of the latest emission data sets available for China. Similar chemical mechanisms have been widely used to study aerosol pollution in China [39,40], demonstrating a high accuracy in the simulation of secondary formation of aerosol. The initial and boundary conditions of pollutants were configured using the global MOZART output.

The WRF-Chem simulation using the above configurations was referred to as the control (CTL) run. To examine the contribution of aerosol transport from external regions, a sensitivity experiment was conducted using the zero-out method [41,42], in which all the anthropogenic emissions of the MEIC in Beijing (marked by the blue square in Figure 1b) were adjusted to zero. The region of interest (ROI) covered all the built-up area of Beijing (Figure 1b). Hereafter, the sensitivity experiment is referred to as the blank (BLK) run, in which the contribution of external transport to the aerosol concentration in Beijing can be estimated. Note that in the remaining sections, the simulation results shown are derived from the CTL run, unless otherwise indicated.

## 3. Results and Discussion

In this section, the WRF-Chem simulations are first evaluated using the wind-profiler measurements and near-surface observations in Beijing, and then the impacts of large-scale synoptic forcings and local PBL structure/process on PM_2.5_ pollution during the studied period are analyzed.

### 3.1. Validation of Simulation Results

**Figure 2:**
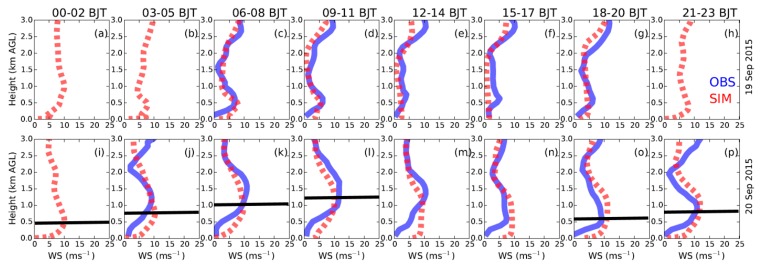
Three-hourly averaged profiles of wind speed (left to right) from 00:00 to 23:00 h Beijing time (BJT) derived from wind-profiler observations (in blue) and WRF-Chem simulations (in red) in Beijing on (**a**–**h**) 19 September and (**i**–**p**) 20 September 2015. The locations of jet nose are marked by the black lines based on the simulated profiles. The simulations are derived from the nearest grid point to the wind-profiler site.

**Figure 3:**
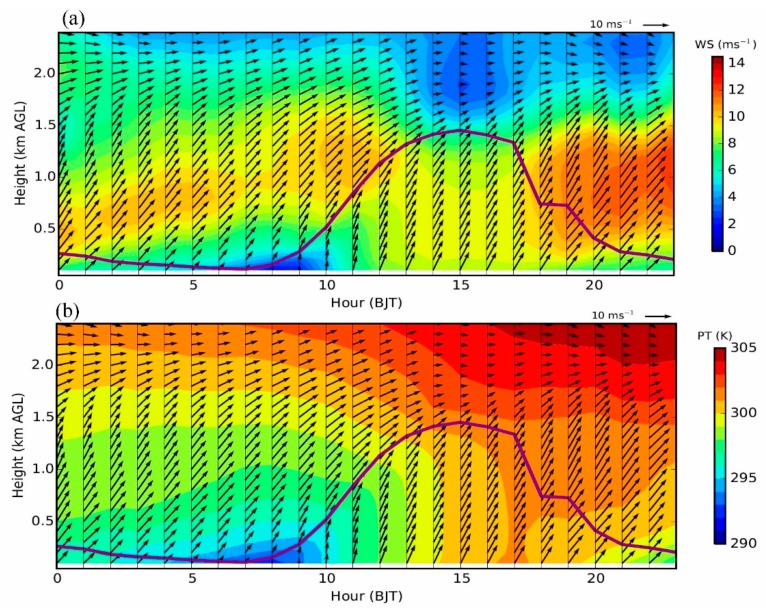
Time-height cross sections showing simulated (**a**) wind speed (WS) and (**b**) potential temperature (PT) in Beijing on 20 September 2015, overlaid with horizontal wind vector fields. The simulated planetary boundary layer (PBL) height is presented as the solid purple line, which is derived using the bulk Richardson number approach. The simulations are derived from the nearest grid point to the wind-profiler site.

In Figure 2, the simulated wind profiles in Beijing are compared with the wind-profiler data. As the measured profiles on 20 September show (Figure 2i–p), the jet nose was observed to occur before sunrise, then gradually weaken and disappear during the afternoon, and strengthen again after sunset. Such a variation of the LLJ was generally well reproduced by WRF-Chem (Figure 2), although the model tended to overestimate the wind speed below 500 m AGL. The occurrence of the LLJ is related to the evolution of PBL thermal structure (Figure 3a,b). In the morning before the establishment of convective boundary layer, the LLJ formed above the PBL. Then, during the afternoon (i.e., from 12:00 to 17:00 h BJT), as the PBL was higher than 1.2 km AGL, the LLJ was weakened and destroyed due to the turbulent mixings within the PBL. After sunset, as the PBL became stable and shallow (Figure 3b), the LLJ redeveloped (Figure 3a). Such co-variations in LLJ and PBL structure are generally in agreement with the theory of Blackadar [26].

**Figure 4:**
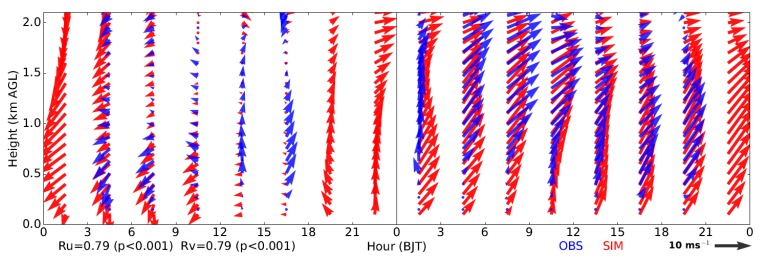
Time-height cross section showing the observed (in blue) and simulated (in red) profiles of horizontal wind vectors in Beijing from 19 to 20 September 2015. The correlation coefficients (i.e., Ru and Rv) of simulated and observed wind components (i.e., u and v, respectively) are also given. The simulations are derived from the nearest point to the wind-profiler site.

In addition, during the studied period, a pronounced transition of wind direction was observed in the lower troposphere, which turns from the northeasterly winds at ~03:00 h BJT on 19 September to the southwesterly winds at ~03:00 h BJT on 20 September (Figure 4). Such a change of wind direction is also accurately simulated by the model. In addition to the wind-profiler data, the simulated profiles of potential temperature and wind were also compared with the radiosonde observations in Beijing, and good agreements were found (Figure S3).

**Figure 5:**
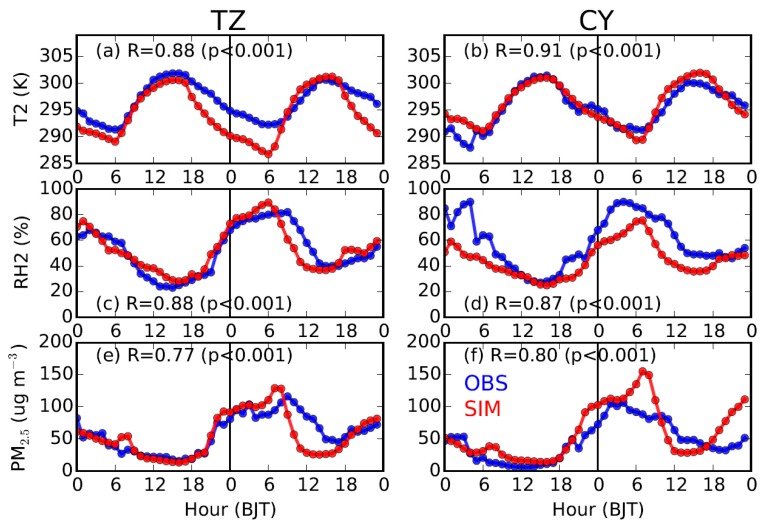
Time series of observed (in blue) and simulated (in red) (top to bottom) 2-m temperature, 2-m relative humidity (RH2), and near-surface PM_2.5_ concentration at (**left**) Tongzhou (TZ) and (**right**) Chaoyang (CY) stations from 19 to 20 September 2015. The correlation coefficients (R) between the simulations and observations are also shown for each panel.

On the ground level, the simulations of 2-m temperature, RH and PM_2.5_ concentration were also validated against the observations (Figure 5). The diurnal variations of both temperature and RH were both well simulated (Figure 5a–d), with correlation coefficients greater than 0.87 (*p* < 0.001). The biases in the temperature and RH at Tongzhou (TZ) and Chaoyang (CY) may be induced by the land-use data, which has certain uncertainties in the urban parameters/areas [43]. Comparing the satellite images of land surface in September 2012 with those in September 2015 (https://worldview.earthdata.nasa.gov), significant differences could be observed. Thus, an accurate and updated description of the land-use in Beijing may improve the model performance [43,44].

With respect to the PM_2.5_ concentration, the diurnal variations and different daily pollution levels during the studied period were also generally well simulated (R ≥ 0.77, *p* < 0.001), although discrepancies existed. The model tends to overrate the PM_2.5_ concentration in the early morning, and underestimate at noon, which may be caused by the constant emission configuration of that MEIC so that the diurnal variation and vertical distribution of emissions were not considered. Comparing CY with TZ, the former is closer to the center of city (Figure 1b) and likely to be influenced by higher emissions; as a result, the peak value of simulated PM_2.5_ concentration at CY is higher than that at TZ.

Overall, the simulated wind profiles, thermal structure, near-surface temperature, RH, and PM_2.5_ concentration in Beijing are generally consistent with the observations, which provides a good basis to use the model outputs to understand the underlying physical processes.

### 3.2. Large-Scale Synoptic Conditions

**Figure 6:**
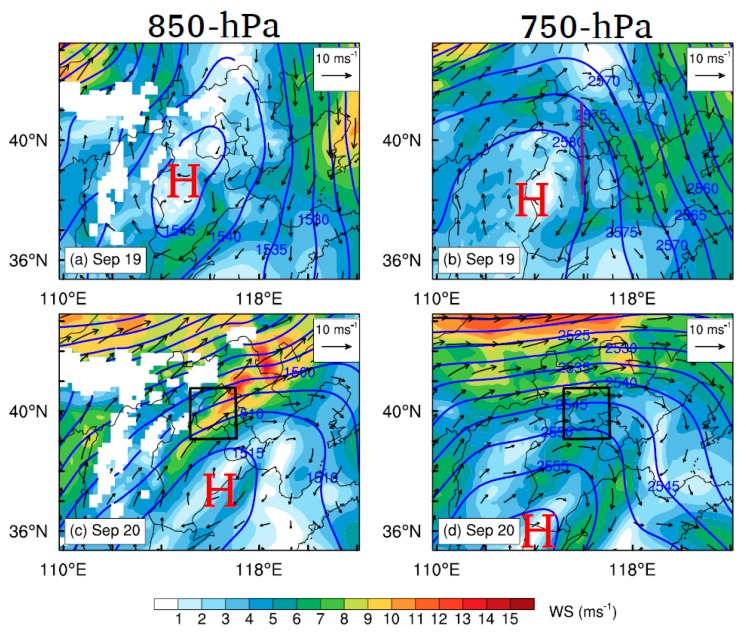
Simulated geopotential height (blue lines) and wind speed (color shaded) fields at (left) 850-hPa and (right) 750-hPa level at 11:00 h BJT on (**a**,**b**) 19 September and (**c**,**d**) 20 September 2015. The text “H” marks the approximate locations of the high pressure system. The violet solid line in (**b**) denotes the locations of vertical cross sections shown in Figure 7.

**Figure 7:**
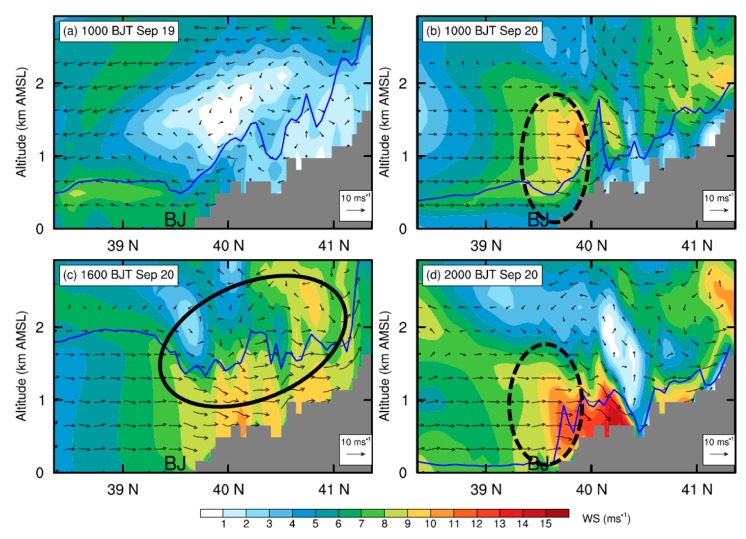
Vertical cross sections of simulated horizontal wind speed (WS) at (**a**) 10:00 h BJT on 19 September, and (**b**–**d**) 10:00, 16:00, and 20:00 h BJT on 20 September, overlaid with the wind vector fields. The locations of the PBL top are marked by the blue lines for each panel. The black solid circle in (**c**) illustrates the approximate locations of mountain-plain breeze circulation, and the black dashed circles in (**b**) and (**d**) denote the locations of the LLJ. Note that the vertical velocity is multiplied by a factor of 10 when plotting the wind vectors, and the approximate location of Beijing is indicated using “BJ”. The locations of the cross section are marked by the violet line in Figure 6b.

The geopotential height fields at 850-hPa level are shown in Figure 6a,c. The transitions of wind direction in Beijing from 19 to 20 September are primarily driven by the day-to-day variations of synoptic forcings. In the morning of 19 September, a high pressure system is located to the southwest of the Hebei province at the 850-hPa level (Figure 6a), which moves easterly and relocates to the southeast of the Hebei province on 20 September (Figure 6c), supporting southwesterly prevailing winds over Beijing (Figure 7b). In the vertical dimension, the pressure gradient across Beijing is stronger at the 850-hPa level than upper levels (e.g., 750-hPa) on 20 September (Figure 6c,d), which favors the formation of jet nose at ~1.5 km AGL in the morning and evening (Figure 2, Figure 3, Figure 4 and Figure 7b). Compared with the climatological study of LLJs in Beijing [24], the synoptic condition on 20 September shows one of typical patterns associated with LLJs in Beijing. From September 2015 to December 2016, around 22.0% of LLJs in Beijing developed under the similar synoptic conditions [24].

### 3.3. Impacts of PBL Structure and LLJ on Pollution 

**Figure 8:**
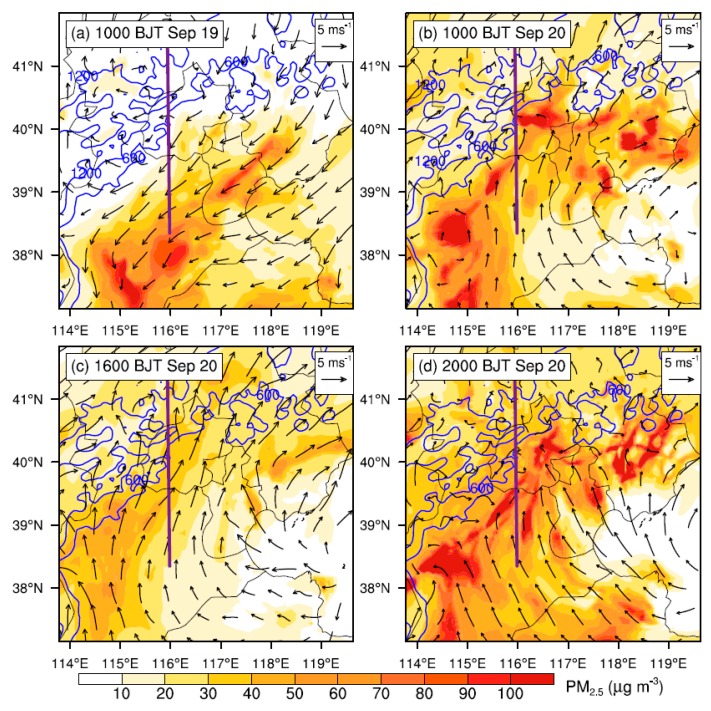
Spatial distributions of simulated near-surface PM_2.5_ concentration at (**a**) 10:00 h BJT on 19 September, and (**b**–**d**) 10:00, 16:00, and 20:00 h BJT on 20 September, overlaid with 10-m wind vector fields. The blue contour lines show the map of terrain height (m AMSL), and the violet line across Beijing from south to north denotes the locations of vertical cross sections shown in Figure 9, Figure 11 and Figure S4.

**Figure 9:**
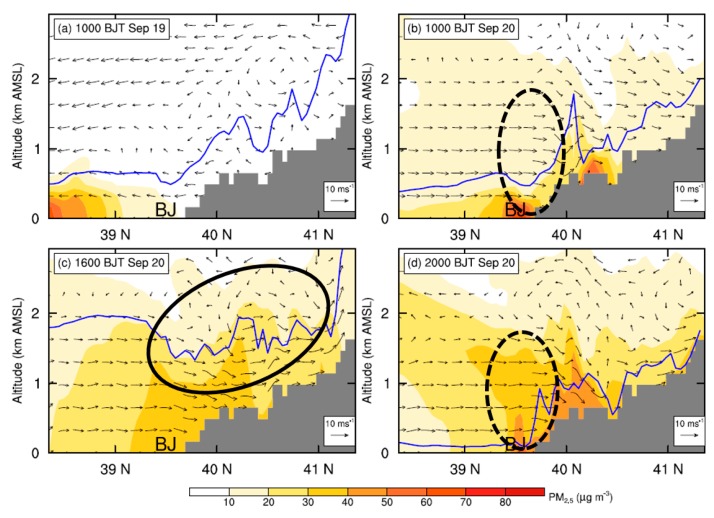
Similar to Figure 7, but for the vertical cross sections of simulated PM_2.5_ concentration at (**a**) 10:00 h BJT on 19 September, and (**b**–**d**) 10:00, 1600, and 20:00 h BJT on 20 September, overlaid with the wind vector fields.

In Figure 7a,b, the vertical sections of meridional wind speed across Beijing on 19 and 20 September are compared. On 19 September, it is the northerly winds dominating over Beijing within the PBL (Figure 4 and Figure 7a), which bring aerosols from Beijing to the downstream southern regions (Figure 8a and Figure 9a). As a result, aerosols are accumulated in the southwest of Hebei while a relatively low concentration is found in Beijing (Figure 8a, Figure 9a). In contrast, on 20 September, the occurrence of southerly prevailing winds (Figure 7b) favors the transport of pollutants from the south Hebei to Beijing, resulting in a high PM_2.5_ concentration in Beijing (Figure 8b–d, Figure 9b–d and Figure S1). Such a spatial distribution of simulated aerosols is generally consistent with the MODIS AOD retrievals shown in the supplementary (Figure S1b,d).

**Figure 10:**
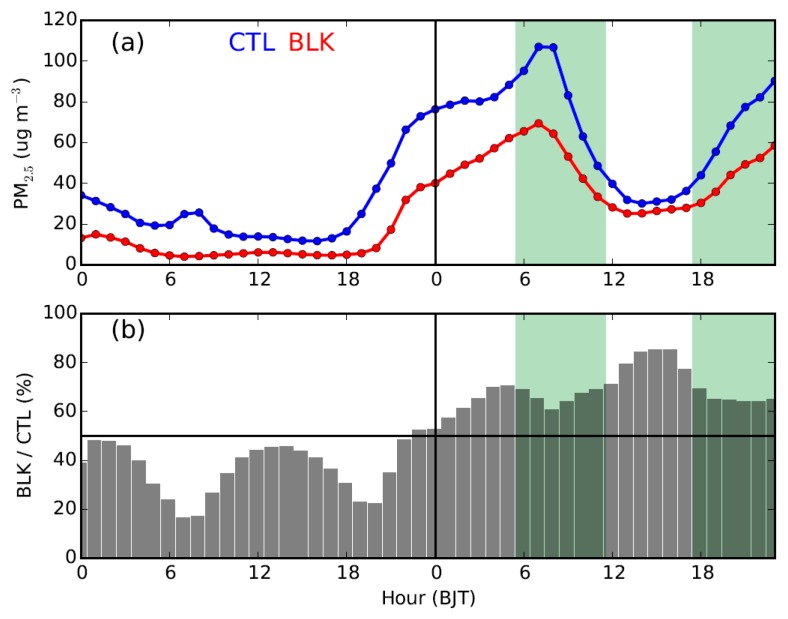
(**a**) Time series of near-surface PM_2.5_ concentrations in Beijing derived from the CTL run (in blue), and those contributed by the anthropogenic emissions outside Beijing (i.e., the BLK run, in red), and (**b**) relative contribution of PM_2.5_ concentration (BLK/CTL) from external regions. The simulations presented here are derived from the urban areas of the ROI denoted by the blue square in Figure 1b. The green shaded areas indicate the approximate periods when the PBL over Beijing is influenced by the LLJ above.

**Figure 11:**
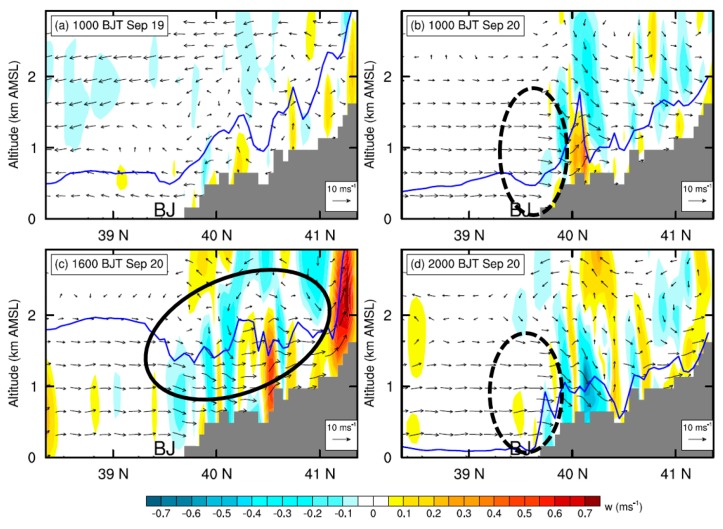
Similar to Figure 7, but for the vertical cross sections of simulated vertical velocity (w) at (**a**) 10:00 h BJT on 19 September, and (**b**–**d**) 10:00, 1600, and 20:00 h BJT on 20 September, overlaid with the wind vector fields.

**Figure 12:**
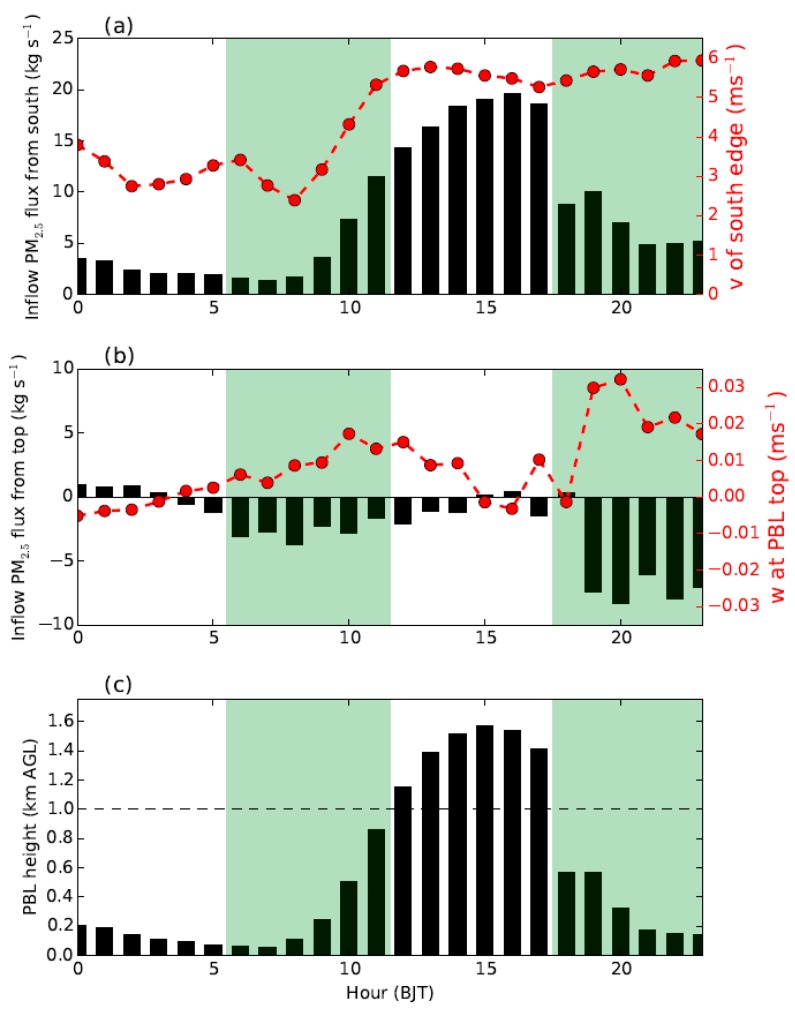
Time series of (**a**) inflow of the PM_2.5_ flux (black bars) within the PBL from south to the ROI in Beijing and the average v-component of horizontal wind (in red) on the south edge; (**b**) inflow of the PM_2.5_ flux (black bars) from top to the ROI and the average vertical velocity (w, in red) at the PBL top level; and (**c**) the average PBL height over the ROI. The locations of ROI are denoted by the blue square in Figure 1b. The green shaded areas indicate the approximate periods when the PBL over Beijing is influenced by the LLJ above.

Comparing the simulated PM_2.5_ concentrations in Beijing derived from the CTL run with those of the BLK experiment (Figure 10), it is found that on 20 September the external transport contributes ~68% (44 μg m^−3^) of PM_2.5_ on average. During that day, the relative contribution of external transport is relatively high in the afternoon (≥80%). Along with the well development of PBL, the intensive momentum exchanges between PBL and upper free troposphere would lead to a stronger southerly wind and transportation of aerosols in the afternoon (Figure 3 and Figure 7c). Meanwhile, the mountains could thermally induce upslope winds along the sloping terrain (Figure 11c and Figure S4c), leading to a closed circulation there. These upslope winds could be superimposed onto the southerly prevailing winds, facilitating the transport of aerosols from south Hebei to Beijing (Figure 8c and Figure 9c). To further understand these processes, the inflow flux of PM_2.5_ from south to the ROI of Beijing (marked by the blue square in Figure 1b) is calculated. The PBL over the ROI is treated as a box. On the south edge, the inflow flux of PM_2.5_ within the PBL could reach ~20 kg s^−1^ in the afternoon (Figure 12a), due to the well development of PBL and resultant strong southerly winds (Figure 12a,c).

In the early morning and late evening, the shallow PBL was decoupled from the free troposphere due to the strong thermal stratification (Figure 3b), leading to weak PBL winds in Beijing (Figure 3a). As a result, the relative contribution of external transport became lower during these periods than in the afternoon (Figure 10b). Coincidently, the LLJ formed above the PBL in Beijing during the morning and the nighttime (Figure 3 and Figure 7b–d). As the vertical motions show in Figure 11b,d, the presence of LLJ over Beijing could induce vertical exchanges of momentum between PBL and upper levels on a local scale [23]. The inflow flux of PM_2.5_ at the PBL top level over the ROI of Beijing is also calculated and shown in Figure 12b. After 18:00 h BJT, along with the drop of PBL height (Figure 12c), a strong vertical motion was induced by the LLJ at the PBL top, which favored the vertical dilution of pollutants in Beijing (Figure 12b). A similar process also occurred in the morning from 05:00 to 11:00 h BJT, but less prominently.

## 4. Conclusions

This study investigated the impacts of large-scale synoptic conditions and local PBL structure/process on PM_2.5_ pollution in Beijing from 19 to 20 September 2015, using wind-profiler data, radiosonde measurements, near-surface meteorological observations, aerosol measurements, and three-dimensional simulations of WRF-Chem.

During the studied period, influenced by southeast-to-northwest pressure gradients across Beijing at the 850-hPa level, the large-scale southerly prevailing winds would favor the transport of aerosols from the southern polluted regions to Beijing, which could contribute to ~68% of the PM_2.5_ measured in Beijing on that day. During that day, the relative contribution of external transport was high in the afternoon (≥80%), which was related to the diurnal evolution of PBL winds. In the afternoon when the PBL was well developed, the intensive momentum exchanges between PBL and upper free troposphere led to a strong southerly wind within the PBL. Meanwhile, the thermally induced upslope winds could superimpose onto the southerly prevailing winds to enhance the transport of aerosols to Beijing in the afternoon.

Besides, influencing by both the large-scale pressure field and local PBL structure, the southerly LLJ developed over Beijing on 20 September, demonstrating prominent diurnal variations. The LLJ formed in the morning before the full establishment of convective boundary layer. During the daytime, as the PBL well developed, the turbulent mixings weakened the LLJ. After sunset, the LLJ strengthened again along with the occurrence of nocturnal boundary layer. The occurrence of the LLJ could enhance the dilution capacity of aerosols over Beijing to some extent, which can lower the aerosol concentration within PBL on a local scale. Finally, it should be noted although this study emphasizes the import roles of physical processes in pollution in Beijing, the influences of chemical formation/reaction also cannot be ignored.

## Figures and Tables

**Table 1:** Technical specifications of the CFL-16 profiler.

Parameters	Values
Direction accuracy	≤10°
Speed accuracy	1 ms^−1^
Vertical resolution	120 m
Lowest level	150 m AGL
Maximum height	16 km AGL
Operating frequency	445 MHz
Aperture	100 m^2^
Gain	33 dB
Peak power	23 kW
Pulse width	0.8 μs
Averaging time	6–60 min

## Supplementary Materials

The following are available online at http://www.mdpi.com/1660-4601/16/4/616/s1. Figure S1: (a, b) The MODIS/Terra RGB (Band 4, 3, 1) true color images and (c, d) spatial distributions of aerosol optical depth (AOD) retrieved from MODIS/Terra at ~10:30 h BJT on (left) 19 and (right) 20 September 2015. The locations of Beijing, Tianjin, and Hebei were denoted by the texts “BJ”, “TJ” and “HB”, respectively, Figure S2: Spatial distribution of PM_2.5_ emissions of September 2012 in North China, provided by Tsinghua University (http://www.meicmodel.org/), Figure S3: Vertical profiles of potential temperature (PT) and wind vector in Beijing at 08:00 h BJT on (a, c) 19 September and (b, d) 20 September, deriving from radiosonde observations (in blue) and simulation results (in red). The simulations are derived from the nearest grid point to the radiosonde station in Beijing, Figure S4: Vertical sections of simulated potential temperature (PT) across Beijing from south to north at (a) 10:00 h BJT on September 19, and (b–d) 10:00 h BJT, 16:00 h BJT and 20:00 h BJT on September 20, overlaid with the wind vector fields. The locations of PBL top are marked by the blue lines for each panel. The black solid circle in (c) illustrates the approximate locations of mountain-plain breeze circulation. Note that the vertical velocity is multiplied by a factor of 10 when plotting the wind vectors, and the approximate location of Beijing is indicated using “BJ”.
