# Supplementary material for: Influence of Boundary Layer Structure and Low-Level Jet on PM2.5 Pollution in Beijing: A Case Study
Source: Int J Environ Res Public Health. 2019 Feb 20;16(4):616. doi: 10.3390/ijerph16040616 (PMC6406672; doi:10.3390/ijerph16040616)
Supplement: Supplementary file 1 [file ijerph-16-00616-s001.pdf]

International Journal of
Environmental Research and Public Health

MDPI

Supplementary Material

Influence of Boundary Layer Structure and Low-Level Jet on $PM_{2.5}$ Pollution in Beijing: A Case Study

Yucong Miao, Shuhua Liu, Li Sheng, Shunxiang Huang and Jian Li

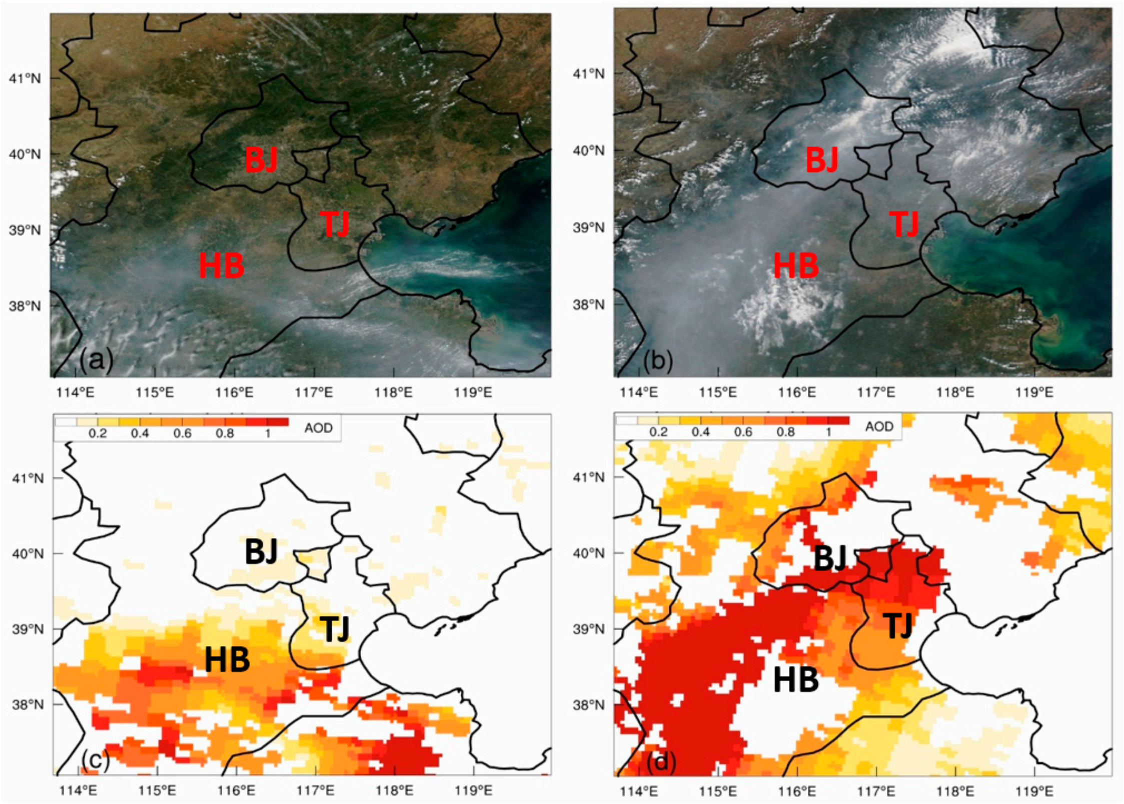

Figure S1. (a, b) The MODIS/Terra RGB (Band 4, 3, 1) true color images and (c, d) spatial distributions of aerosol optical depth (AOD) retrieved from MODIS/Terra at ~10:30 h BJT on (left) 19 and (right) 20 September 2015. The locations of Beijing, Tianjin, and Hebei were denoted by the texts "BJ", "TJ" and "HB", respectively.

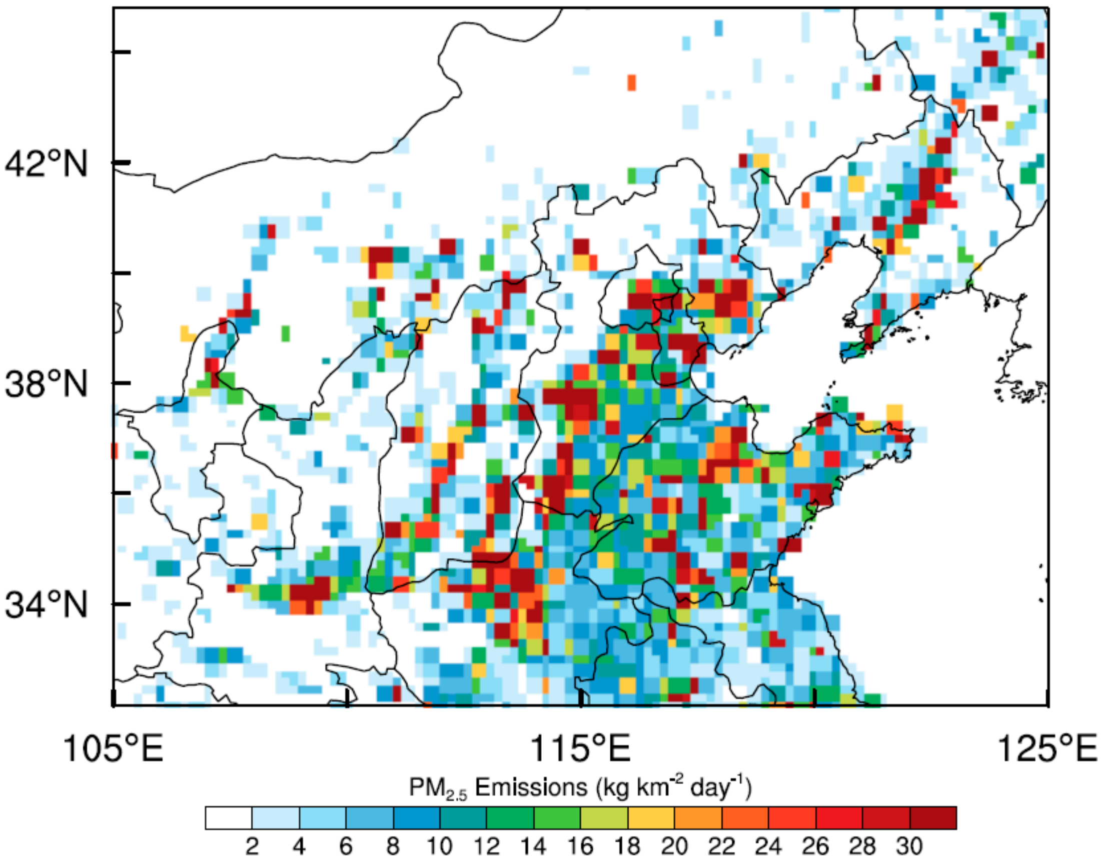

Figure S2. Spatial distribution of $PM_{2.5}$ emissions of September 2012 in North China, provided by Tsinghua University (http://www.meicmodel.org/).

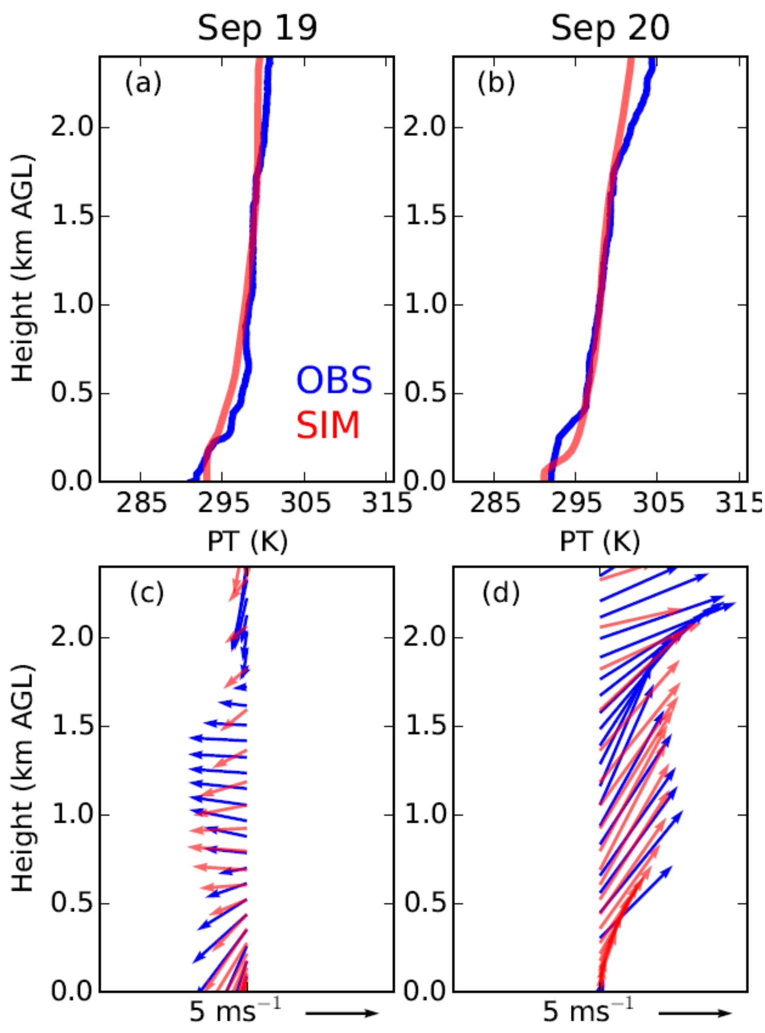

Figure S3. Vertical profiles of potential temperature (PT) and wind vector in Beijing at 08:00 h BJT on (a, c) 19 September and (b, d) 20 September, deriving from radiosonde observations (in blue) and simulation results (in red). The simulations are derived from the nearest grid point to the radiosonde station in Beijing.

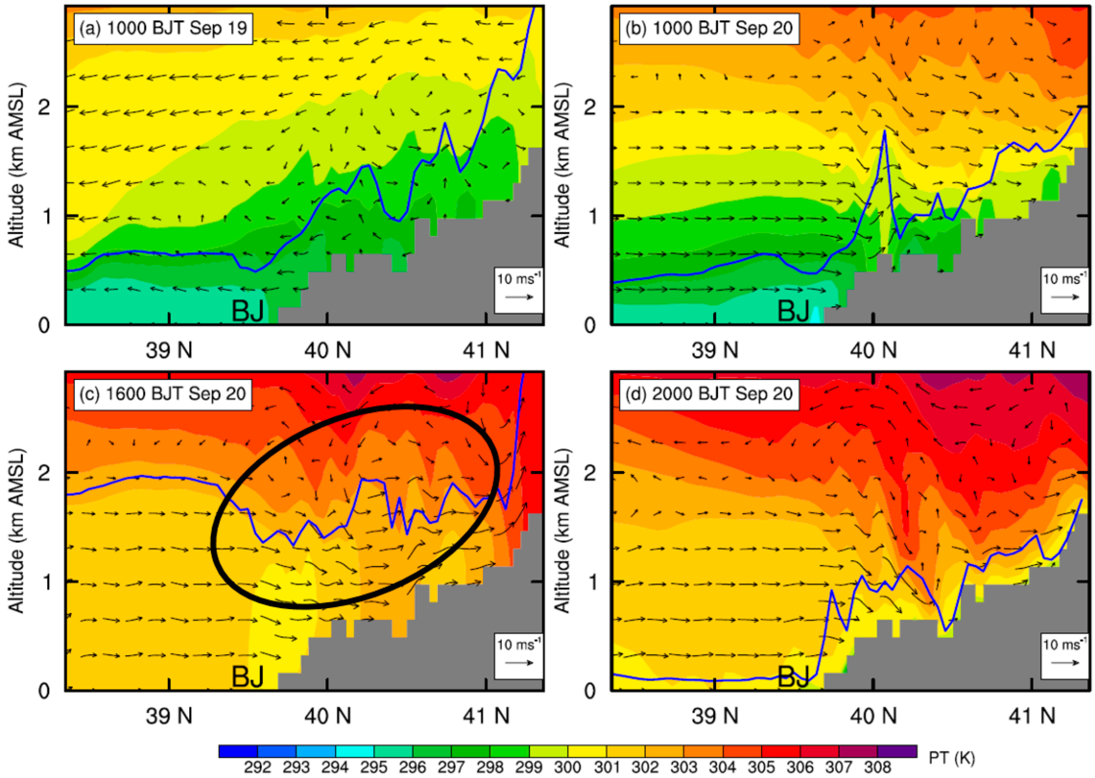

Figure S4. Vertical sections of simulated potential temperature (PT) across Beijing from south to north at (a) 10:00 h BJT on September 19, and (b-d) 10:00 h BJT, 16:00 h BJT and 20:00 h BJT on September 20, overlaid with the wind vector fields. The locations of PBL top are marked by the blue lines for each panel. The black solid circle in (c) illustrates the approximate locations of mountain-plain breeze circulation. Note that the vertical velocity is multiplied by a factor of 10 when plotting the wind vectors, and the approximate location of Beijing is indicated using "BJ".

© 2019 by the authors. Submitted for possible open access publication under the terms and conditions of the Creative Commons Attribution (CC BY) license (http://creativecommons.org/licenses/by/4.0/).
